# High-throughput identification of bacterial β-glucuronidase inhibitors using machine learning

**DOI:** 10.1080/19490976.2026.2681789

**Published:** 2026-06-05

**Authors:** Bohan Zhang, Haoran Yue, Anna Skalse, Nannapat Sangfuang, David Shorthouse, Simon Gaisford, Abdul W. Basit

**Affiliations:** a Department of Pharmaceutics, UCL School of Pharmacy, London, UK

**Keywords:** Artificial intelligence, bacterial enzyme, beta-glucuronidase, biopharmaceuticals, drug degradation, drug metabolism, gut microbiome, inhibitor prediction, machine learning, RDKit fingerprints, SMILES

## Abstract

The human gut microbiome plays a vital role in regulating host physiological functions and influencing the pharmacokinetics of interventions, particularly drug metabolism, which in turn affects pharmacodynamics. Gut microbial β-glucuronidase (GUS) is a key bacterial enzyme that modulates drug therapeutic outcome and gastrointestinal toxicity through deconjugating glucuronidated drug metabolites. Despite this, systematic high-throughput prediction of GUS inhibitors remains limited by sparse experimental data and the translational shortcomings of known compounds. Here, machine learning is applied as a powerful tool to identify potential GUS inhibitors from more than 10,000 FDA-approved drugs, food additives, and excipients. In this study, both unsupervised and supervised machine learning models were trained on literature-derived data describing the inhibitory potency of 122 compounds against *Escherichia coli* GUS (EcGUS). These models were compared with a newly developed SMILES-based 1D feature-embedded, self-attention classification model (IC-tf) designed for high-throughput screening. To improve interpretability, a dual-level analysis that combines SHAP attribution of handcrafted descriptors with branch-level transformer attention was applied to the IC-tf model. All models demonstrated strong predictive performance, with ROC-AUC values of 85.9%–89.3% under 3-fold cross-validation, with the IC-tf model showing the highest predictive power. *In vitro* validation with an external set of 20 compounds confirmed strong predictive accuracy for the Random Forest and IC-tf models. This work establishes a scalable computational framework for high-throughput discovery of gut microbial GUS inhibitors, facilitating efficient screening of co-administered drugs and excipients with the translational potential to improve drug bioavailability and reduce gastrointestinal toxicity.

## Graphical abstract

**Figure:**
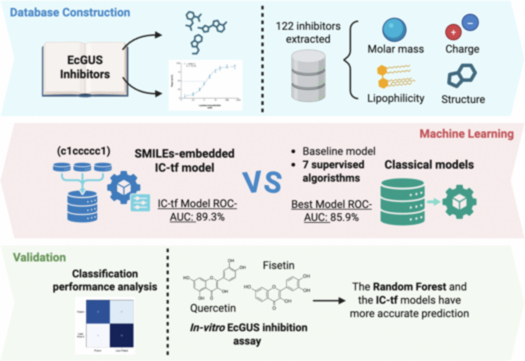

## Introduction

The human gut microbiome constitutes a highly complex ecosystem that mediates interactions between the host and the environment. Increasing evidence highlights the intricate relationship between the gut microbiome and commonly used non-antibiotic drugs.^1^ The microbiome can modulate an individual's drug response by enzymatically transforming drug structures, thereby altering their bioavailability, bioactivity, or toxicity. Numerous drugs have now been identified to be susceptible to microbial metabolism, which can lead to reduced bioavailability.^2^ This metabolic interference poses significant challenges for the design of extended-release and targeted-release formulations, complicating efforts to achieve effective once-daily dosing or localized treatment of gastrointestinal (GI) diseases.^3^ One of the drug metabolism-related enzymes expressed by the gut microbiota is the β-glucuronidase (GUS), which naturally catalyses the breakdown of carbohydrates and glycoconjugates (e.g., peptidoglycans, glycosides, glycolipids), as well as the deconjugation of small-molecule natural glucuronides and glucuronidated metabolites of endogenous and xenobiotic compounds generated by host phase II UDP-glucuronosyltransferases (UGTs).^4^ These enzymes are widely distributed across multiple bacterial phyla, including *Firmicutes*, *Bacteroidetes*, *Verrucomicrobia*, and *Proteobacteria*, which are typically found in human gut microbiota.^5^ Through the deconjugation activity, GUS influences the metabolism of drugs, dietary constituents, and endogenous compounds such as conjugated estrogens.^6^ Previous studies have demonstrated that the gut microbial GUS promotes certain drug-induced GI toxicities resulting from the reversal of phase II glucuronidation. For example, the anticancer drug irinotecan can cause severe diarrhea when its glucuronide conjugate is deconjugated to the toxic metabolite SN-38 by microbial GUS in the GI tract.^7^ Moreover, inhibition of gut microbial GUS has been shown to protect against intestinal injury and reduce dose-limiting toxicities associated with other agents, such as regorafenib.^8^ In this context, inhibition of bacterial GUS represents a promising strategy to mitigate drug-induced toxicity, and the screening of potent gut microbial GUS inhibitors is highly desirable.

Several studies have investigated the inhibition of gut microbial GUS using the well-documented model of *Escherichia coli* GUS (EcGUS) as a representative. Potent inhibitors such as UNC10201652 and certain pyrazolo[4,3-c]quinoline derivatives have been identified, with reported IC₅₀ values of 0.1 and 0.03 µM, respectively.^9^ ^,^ ^10^ However, most of these known inhibitors were derived from structurally related molecules originating from a single natural scaffold with unknown effects in humans, and none have demonstrated evidence or sufficient potential to improve drug bioavailability upon co-administration with susceptible drugs. Furthermore, among more than 3000 FDA-approved small-molecule drugs and more than 10,000 food additives and drug excipients, only a few have been tested for inhibitory effect against EcGUS. Therefore, high-throughput approaches for the identification of potent gut microbial GUS inhibitors are still needed, particularly to enable efficient screening and prediction of inhibitors from lists of molecules of interest. Recently, numerous machine learning (ML) and deep learning (DL) approaches have been applied to predict the inhibition of drug-metabolizing enzymes, such as cytochrome P450 enzymes.^11^ These studies typically rely on molecular descriptors computed from Simplified Molecular-Input Line-Entry System (SMILES) notations and have achieved high predictive accuracy, highlighting their potential for identifying inhibitors to improve drug bioavailability. In addition, advanced DL techniques have been directly applied to SMILES representations to enhance inhibitor prediction. For instance, Grimberg et al. utilized convolutional neural network (CNN) architectures to process SMILES strings for the prediction of small-molecule inhibitors targeting RNA.^12^ The previous research showed the immense potential of applying ML to identify inhibitors for certain enzymes, and this study aims to develop ML and DL programs to find potential inhibitors for EcGUS to pave the way for more efficient treatment and more available co-administration.

In this study, a range of classical ML strategies was developed and compared for their ability to predict the potency of EcGUS inhibitors, using the Half-maximal inhibitory concentration (IC_50_) values of known inhibitors as input. In addition, a DL model was constructed that integrated ChemBERTa-derived SMILES string embeddings with RDKit-generated molecular descriptors. The training dataset was compiled through strategic literature mining, and model performance was benchmarked against a baseline classifier that reported the arithmetic mode (i.e., the most frequent potency class) of the training set. The best-performing models were subsequently evaluated on an independent dataset to assess predictive behavior against unseen data, supported by in vitro inhibition assays. The results highlight the advantage of combining SMILES-derived 1D features with 2D molecular descriptors, demonstrating the potential of these models to facilitate the prediction of EcGUS inhibitory potency for untested compounds.

## Materials and methods

### Materials

Quercetin dihydrate was purchased from MP Biomedicals, LLC (California, USA). Fisetin was purchased from Cambridge Biosciences (Cambridge, UK). P-Nitrophenyl-β-d-glucuronide acid (PNPG), *E. coli* β-Glucuronidase (EcGUS) and Phosphate buffered saline (PBS) tablet were all purchased from Sigma Aldrich (Dorset, UK). Dimethyl sulfoxide (DMSO) was purchased from Thermofisher Scientific (Massachusetts, US).

### Database preparation

#### Data sourcing

The *E. coli* beta-glucuronidase inhibitor training database was constructed by retrieving the inhibitor chemical structures and biological results from the literature, identified via PubMed and Web of Science Core Collection. The inhibitors identified in the literature studies were tested against *E. coli* beta-glucuronidase in vitro using p-nitrophenyl-β-glucopyranoside (pNPG) as the substrate. IC_50_ values reported in the literature were recorded as an index of inhibitory efficacy, as the most widely used and informative measure of an inhibitor's efficacy.^13^

**Figure 1.**
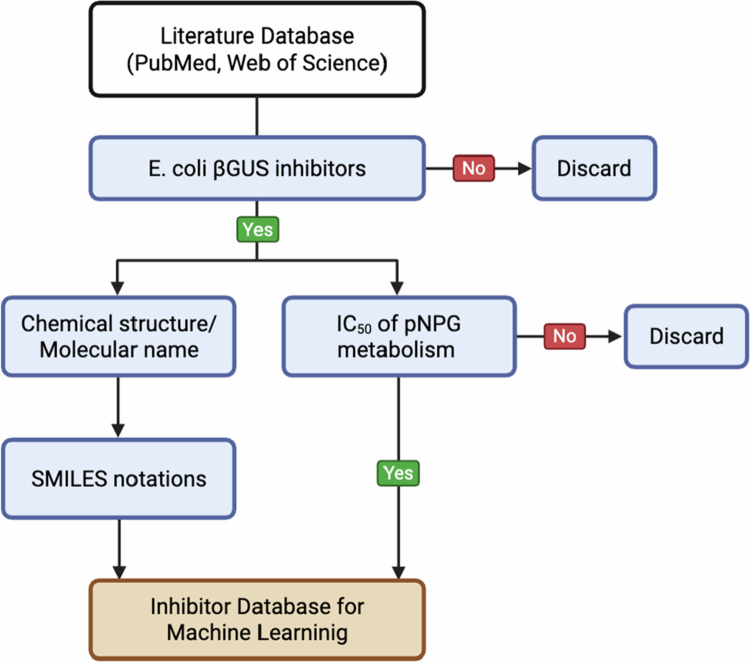
Data collection flowchart for constructing the *E. coli* beta-glucuronidase inhibitor database.

Specific search terms were used to find literature studies identifying inhibitors of *E. coli* beta-glucuronidase. The primary search terms included “bacteria”, “*E. coli*”, “β-glucuronidase” and “inhibitor” combined using searching operators “AND” and “OR” to refine the results. Table 1 presents the specific search terms used along with the number of studies retrieved for each query. To ensure the relevance and quality of the selected studies, domain experts reviewed each entry prior to its inclusion in the training dataset. The complete process of data selection and extraction is illustrated in Figure 1.

**Table 1.** Search terms used to identify relevant data for the training dataset and the number of studies listed per term.

Search term 1	Search operator	Search term 2	Search operator	Search term 3	No. of PubMed results	No. of WoS results
Bacteria	AND	β-glucuronidase	AND	Inhibitor	290	56
*E. coli*	AND	β-glucuronidase	AND	Inhibitor	112	47
Bacterial β-glucuronidase	AND	inhibitor			184	108
*E. coli* β-glucuronidase	AND	inhibitor			112	47

#### Data extraction, inhibitor featurization, and data pre-processing

Most molecular names and chemical structures of the inhibitors were obtained directly from the literature as raw data. The SMILES notations for the majority of molecules were retrieved from the PubChem database.^14^ SMILES has been widely validated as a reliable format for quantitative structure–activity relationship (QSAR) analyses^15^ and has been previously applied in machine learning-based inhibitor prediction models.^16^ However, in some cases, the literature provided only graphical representations of chemical structures without corresponding names, or the molecules were not available in the PubChem database. For these compounds, SMILES notations were manually generated by inputting the structures into the online SMILES generator developed by Cheminfo.^17^ Following this initial retrieval, a rigorous curation protocol was applied. Compounds with missing, ambiguous, or non-standard IC_50_ values, as well as those lacking clear experimental conditions, were excluded from the dataset. For duplicate compounds reported across multiple publications, a single representative entry was retained if the IC_50_ values were consistent. In cases where conflicting IC_50_ values were reported, the measurements obtained under comparable experimental conditions and also with clearly defined and standardized experimental protocols were prioritized. Following data extraction, a structured database was constructed, with each entry including the molecule name, SMILES notation, and corresponding IC₅₀ value.

To facilitate model learning, inhibitory efficacy values were converted into a binary classification: “Potent” (IC₅₀ ≤ 1 µM) and “Less Potent” (IC₅₀ > 1 µM). The threshold of 1 µM for defining potent inhibitors in drug development, delivery and quantitative structure-activity relationship (QSAR) models is supported by the literature.^18-20^ After preliminary data processing, molecular featurization was performed to encode each compound with a set of chemically diverse descriptors. Specifically, 208 physicochemical properties were computed for each molecule using RDKit (version 2022.09.05) based on their isomeric SMILES notations. A comprehensive list of these features is available in RDKit’s documentation (https://www.rdkit.org/), and the feature names with corresponding index codes used in this study are summarized in Table S3. Furthermore, the binary inhibitory class was label-encoded as 1 for “Less Potent” and 0 for “Potent”. All numerical features were normalized to the range (0, 1) using Equation (1):

(1) $$X_{\mathrm{scaled}}=\;\frac{X-\;X_{\min}}{X_{\max}-\;X_{\min}},$$

where *X* denotes the original feature value, while *X*_min_ and *X*_max_ represent the minimum and maximum values of that feature within the dataset, respectively. All data manipulation and preprocessing were carried out using the Scikit-learn library (version 1.2.2).

### Model development

#### Classical model benchmarking

Several machine learning algorithms that are well-suited for small datasets were developed and evaluated for predicting potent inhibitors.^21^ Among the tree-based models selected were Extra Trees, Decision Tree (DT), Random Forest (RF), and XGBoost. These models operate by making a series of binary (either-or) decisions at successive nodes within a decision tree structure. For linear models, logistic regressions with Lasso (LR_lasso) and Ridge (LR_ridge) penalties were employed. Logistic regression is a classification algorithm that maps continuous input features to categorical outputs using a logistic function. Additionally, k-nearest neighbors (kNN) models were included, with k values set to 1, 2, and 4 (referred to as kNN-1, kNN-2, and kNN-4, respectively). The kNN algorithm classifies unlabeled samples based on their similarity to the nearest labelled examples in the feature space. A more comprehensive overview of these modelling approaches and their applications in drug discovery and development is provided in the systematic review by Vamathevan et al. (2019).^22^ The performance of all models was benchmarked against a baseline model implemented using the DummyClassifier, which consistently predicted the most frequent class in the training dataset, regardless of input features. The Random Forest (RF) model was optimized with the following hyperparameters: n_estimators = 1400, min_samples_split = 2, min_samples_leaf = 1, max_features = “auto”, max_depth = None, criterion = “entropy”, and bootstrap = True. The Decision Tree (DT) model was trained using criterion = “entropy”, max_depth = None, max_features = “log2”, min_samples_leaf = 1, and min_samples_split = 5. All other models were trained using their respective default hyperparameters. Model evaluation was conducted using 3-fold cross-validation (CV), and performance was assessed based on receiver operator characteristic-area under curve (ROC-AUC), balanced accuracy, and the weighted F1 score (F1_weighted).

ROC-AUC is the primary metric used for optimizing machine learning models, as it effectively reflects the model’s ability to distinguish between the binary categories.^23^ ROC works by plotting the true positive rate (TPR) against the false positive rate (FPR), and the equation is presented in Equation (2):

(2) $$\mathrm{AUC}=\;\sum_{i=1}^{n-1}({\mathrm{FPR}}_{i+1}-{\mathrm{FPR}}_{i})\times \frac{{\mathrm{TPR}}_{i+1}+{\mathrm{TPR}}_{i}}{2}.$$

The rest two metrics are particularly well-suited for evaluating performance on imbalanced datasets.^24^ The mathematical formulations for balanced accuracy and the weighted F1 score are provided in Equations (3) and (4), respectively. In this multiclass classification context, true positives (TP), true negatives (TN), false positives (FP), and false negatives (FN) were computed using a one-vs-rest approach.

(3) $$\mathrm{Balanced}\;\mathrm{accuracy}=\;\frac{\frac{\mathrm{TP}}{\mathrm{TP}+\mathrm{FP}}+\frac{\mathrm{TN}}{\mathrm{TN}+\mathrm{FN}}}{2},$$

(4) $$F1_{\mathrm{weighted}}=\frac{\mathrm{TP}}{\mathrm{TP}+\frac{1}{2}(\mathrm{FP}+\mathrm{FN})}.$$

### Feature selection

To assess feature importance, RF, XGBoost and LR_lasso models, which inherently support feature importance analysis, were used to generate feature importance matrices after being trained on the dataset. Feature selection was performed within the training data prior to model evaluation to avoid data leakage. The top 20 most important features identified by each model were recorded. The influence of these selected features on model performance was further evaluated using 3-fold CV, with ROC-AUC, balanced accuracy, and weighted F1 score as evaluation metrics.

### Fusion framework architecture

**Figure 2.**
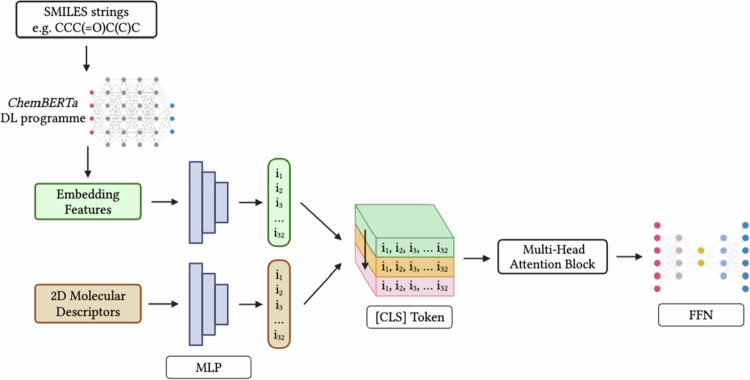
Illustration of the architecture of the SMILES-embedded model, which emphasizes the extraction of informative molecular representations directly from SMILES notations. These embeddings, obtained using a ChemBERTa-based encoder followed by dimensionality reduction through an MLP, capture rich structural information critical for compound classification.

A model that integrates molecular structural representations and expert-selected physicochemical features has been developed through a multi-branch fusion framework (Figure 2). Specifically, SMILES notations are processed using a ChemBERTa-based embedding pipeline, producing a 1D vector containing 784 numerical features.^25^ This vector is then passed through a multi-layer perceptron (MLP) to yield a fixed-length 32-dimensional embedding. In parallel, manually curated physicochemical descriptors are input into a separate MLP to produce a 32-dimensional embedding. The two resulting representations, molecular embeddings and physicochemical feature embeddings, are concatenated and aggregated using a [CLS] token. The unified representation is then passed through a multi-head self-attention module to capture higher-order interactions between features and enhance the model’s discriminative capacity. This self-attention-based classification model, referred to as IC-tf, was trained using 80% of the dataset for training and 20% for testing. The architecture consists of 3 layers of multi-head self-attention, each with 8 attention heads, and a dropout rate of 0.4 was employed to reduce overfitting. The model inputs include both molecular and physicochemical embeddings, each mapped to a 32-dimensional space. Training was conducted over 300 epochs to ensure sufficient convergence. Finally, a feedforward neural network (FFN) serves as the classification head to predict compound activity.

### Model evaluation

To enable a more detailed evaluation of the best-performing models, classification confusion matrices were plotted. The confusion matrices were generated using a simple training-prediction scenario utilizing a standard 80/20 train-test split without applying cross-validation. Predictions on the holdout test set were compared against the corresponding ground truth labels and visualized as heatmaps. Given the moderate class imbalance in the dataset, synthetic data augmentation or resampling was omitted from the primary analysis to prevent the introduction of artificial noise into a limited dataset. Instead, class proportions were preserved using stratified data splitting, and model performance was evaluated using imbalance-aware metrics including ROC-AUC, balanced accuracy, and weighted F1 score. In addition, a cost-sensitive learning analysis using class-weighted training was performed as a supplementary sensitivity analysis (Figure S2).

**Figure 3.**
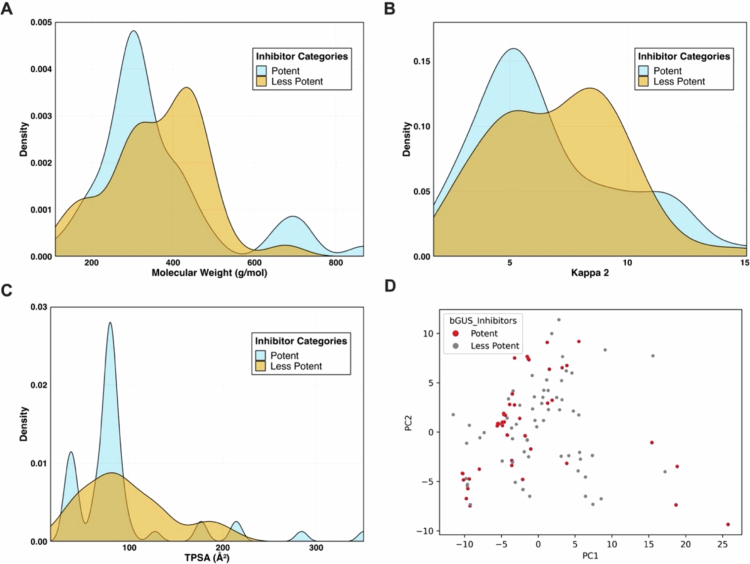
Data distribution of the EcGUS inhibitor database on (A) molecular weight, (B) Kappa 2 and (C) TPSA (topological polar surface area). (D) PCA analysis of the database was also conducted.

To further assess model stability across alternative data partitions, a 5× repeated stratified 3-fold cross-validation was performed with a 60:40 train-test split (FigureS3). Performance distributions across all 15 validation folds were recorded for each model to evaluate robustness under reduced training set sizes. Ultimately, because the limited dataset size necessitates maximizing the available training information for deep learning feature extraction, the predefined 80:20 train-test split was retained as the primary evaluation protocol for IC-tf training and analysis.

### Interpretability analysis

To improve the interpretability of the IC-tf model, a dual-level analysis was performed. At the feature level, SHAP was used to estimate the contribution of individual handcrafted molecular descriptors to model predictions. At the branch level, transformer attention weights were analyzed to assess the relative reliance of the classifier on the handcrafted descriptor branch versus the SMILES-based ChemBERTa embedding branch. Crucially, because the current IC-tf architecture compresses each modality into a single latent token before transformer fusion, attention was interpreted at the branch level rather than at the level of individual descriptors or SMILES tokens. Representative local case studies were further examined by combining branch-level attention with descriptor-level SHAP attributions.

### Enzyme inhibition assays

The inhibitory effects of quercetin and fisetin against EcGUS were investigated by using PNPG as the substrate. Briefly, the incubation mixture with a total volume of 100 μL was composed of 2 μg/mL EcGUS and the test flavonoids in PBS buffer. Following a 5-mi pre-incubation at 37 °C, the reaction was initiated by adding 10 μL PNPG (500 μM, final concentration), with the final concentration of DMSO at 2% (v/v) without affecting the enzymatic activities.^26^ PNPG hydrolysis with or without inhibitor was performed at 37 °C for 30 min. The signal of the hydrolysis product *p*-nitrophenol (PNP) was detected at 405 nm by a CLARIOstar® Plus Microplate Reader (BMG LABTECH, Germany). 2% DMSO was used as a negative control. Blank group without EcGUS was set up to exclude potential absorbance interference by flavonoids during the detection stage. All assays were conducted in triplicate, and the data were shown as mean ± SD.

## Results and discussion

### Database overview and unsupervised model

A total of 290 publications were initially retrieved through search queries on PubMed and Web of Science, as summarized in Table 1. Following refinement using specific keywords and the removal of irrelevant articles, 18 publications were selected for inclusion in the study^9^ ^,^ ^26-42^ (Table S1), comprising one review article and 17 primary research papers. Except for the review paper that reported 43 inhibitors with diverse chemical structures and origins, no single study disproportionately contributed to the dataset, thereby minimizing the potential for bias arising from any individual source. From these sources, 122 unique molecules were extracted and compiled into an inhibitor database. Exploratory analysis revealed that 49 out of 122 molecules exhibited high potency, with IC₅₀ values below 1 µM, while the remaining 73 were classified as less potent. Although no universal IC_50_ threshold has been established for EcGUS inhibitors, the cutoff of 1 µM has been adopted in previous studies.^18^ ^,^ ^19^ In the present work, sensitivity analysis and the distribution of IC₅₀ values across the 122 compounds also suggested that this threshold yields a more balanced binary classification and improves model sensitivities for both RF and XGBoost models (Figure S1, Table S2). While some drugs or additives can reach concentrations exceeding 1 µM in the gut lumen, the use of a relatively stringent threshold prioritizes the identification of highly potent inhibitors. This may be advantageous for downstream applications, as compounds effective at lower concentrations are more likely to translate into practical dosing strategies and facilitate formulation or co-administration.^43^ However, this binary classification approach may also exclude moderately active compounds that could still be effective under physiological conditions, highlighting a trade-off between potency-driven selection and retaining a broader range of potentially active compounds.

Further analyses were conducted focusing on the molecular weight, structure, and polarity of the inhibitors, as previous research has suggested that ligand affinity for the bacterial GUS active site is influenced by these physicochemical properties.^44^ The molecular weight pattern indicated a trend toward more potent inhibitory activity among compounds with drug-like molecular weights around 200–400 g/mol^45^ (Figure 3A). This observation aligns with previous studies showing that *E. coli* β-glucuronidase preferentially interacts with small-molecule glucuronide substrates.^44^ Apart from the molecular weight, the second-order molecular shape characteristic descriptor Kappa 2, which quantifies the degree of flexibility of the bonding pattern based on 2D topology.^46^ Previous studies have shown that EcGUS exhibits a preference for phenolic and fused phenolic substrates, which are characterized by moderate Kappa 2 values.^44^ Analysis of the Kappa 2 index for the 122 inhibitors revealed a similar trend that potent inhibitors are moderately branched with Kappa 2 around 5 instead of being bulky (Figure 3B). Polarity was assessed using the topological polar surface area (TPSA), a descriptor that quantifies the total surface area of polar atoms in a molecule. While the density curves for both potent and less potent inhibitors peaked at similar TPSA values (Figure 3C), potent inhibitors were more concentrated within the 60–80 Å^2^ range. In contrast, less-potent inhibitors displayed a broader distribution of TPSA values.

The exploratory data analysis suggested potential correlations between inhibitor potency and three key physicochemical properties: molecular weight, molecular shape, and TPSA. However, these were just 3 out of 208 physicochemical descriptors used to characterize the inhibitors in the dataset with high complexity and high dimensionality. Given this, a more systematic and scalable approach was required to effectively identify potent EcGUS inhibitors. Machine learning was identified as a suitable solution due to its proven capability in modelling high-dimensional data and uncovering complex relationships.^47^ ^,^ ^48^

Unsupervised modelling was conducted using Principal Component Analysis (PCA), a straightforward linear technique commonly used for visualizing structure–activity relationships (Figure 3D).^47^ Visualization of all 122 data points revealed no clear separation or strong patterns linking the compounds' physicochemical features to their inhibitory potency. This result further underscored the need for supervised learning approaches to achieve more accurate and meaningful predictions.

### Supervised modelling

**Figure 4.**
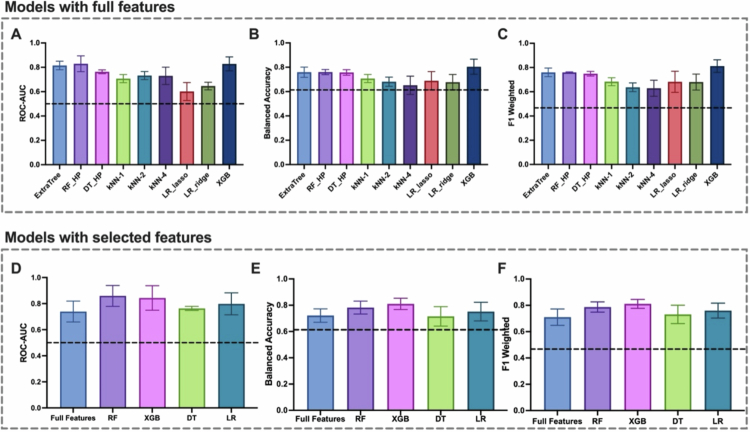
Model performances across three evaluation metrics on the EcGUS inhibitor database with full feature (a–c) or selected features (d–f) as inputs. Dashed lines represent baseline model performance, CV = 3; full results are available in Table S4.

Supervised machine learning techniques were applied to the EcGUS inhibitor database. The complete performance metrics are provided in Table S4, with the top-performing results summarized in Figure 4A–C. All models significantly outperformed the baseline, which had a ROC-AUC of 50%, a balanced accuracy of 61.3%, and a weighted F1 score of 46.7%. Among the tested models, the random forest and XGBoost classifiers achieved the highest ROC-AUC values of 82.9% and 82.8%, respectively. In terms of balanced accuracy and weighted F1 score, the XGBoost model performed best, with a balanced accuracy of 80.4% and a weighted F1 score of 81.2%. In contrast, kNN and logistic regression models did not surpass the performance of the non-linear tree-based models across all three evaluation metrics. These results underscore the superior ability of non-linear algorithms to capture complex relationships between molecular physicochemical properties and inhibitory potency against EcGUS.

To further enhance model performance, feature selection was performed using the best-performing RF and XGBoost algorithms. This process identified the top 20 most influential physicochemical descriptors associated with inhibitor potency, allowing for refinement of the feature set. The selected features are listed in Table 2. Both algorithms identified the electrotopological state (“Estate”) and corresponding van der Waals surface area (“VSA”) of atoms as highly important in inhibitors' potency, which aligned with previous literature.^49^ ^,^ ^50^ In addition to electrotopological descriptors, the algorithms identified specific functional groups that are critical for inhibitory potency. Both algorithms highlighted the presence of a bicyclic structure (*fr_bicyclic*) as an important feature, consistent with previous findings that such structures are commonly found in bacterial β-glucuronidase inhibitors.^51^ Furthermore, the absence of an aromatic hydroxyl group on bicyclic scaffolds has been shown to reduce the IC₅₀ values of EcGUS inhibitors,^26^ aligning with the *fr_Ar_OH* feature selected by the XGBoost algorithm.

**Table 2.** Feature selected by RF and XGBoost algorithms.

RF		XGBoost	
Index	Feature name	Index	Feature name
0	MaxEStateIndex	60	SMR_VSA10
60	SMR_VSA10	98	VSA_EState5
2	MaxAbsEStateIndex	4	qed
97	VSA_EState4	0	MaxEStateIndex
24	BCUT2D_MRLOW	43	Kappa3
159	fr_bicyclic	97	VSA_EState4
81	SlogP_VSA9	69	SlogP_VSA1
10	MaxPartialCharge	159	fr_bicyclic
13	MinAbsPartialCharge	24	BCUT2D_MRLOW
94	VSA_EState10	95	VSA_EState2
98	VSA_EState5	3	MinAbsEStateIndex
78	SlogP_VSA7	22	BCUT2D_LOGPLOW
121	MolLogP	92	EState_VSA9
95	VSA_EState2	130	fr_Ar_OH
41	Kappa1	19	BCUT2D_CHGHI
4	qed	79	SlogP_VSA8
139	fr_NH1	113	NumHAcceptors
16	FpDensityMorgan3	115	NumHeteroatoms
3	MinAbsEStateIndex	41	Kappa1
113	NumHAcceptors	10	MaxPartialCharge

The machine learning was repeated using the refined features with the same modelling and hyperparameters. After narrowing down the features, the RF and XGBoost models using only the first 3, 5, 10, 15, 17 or 20 features from the selected list were performed. Both models with 20 features achieved the best performance across all three evaluation metrics (Figure S4) and are better than the previous models using the full 208 features (Figure 4D–F). Refined RF model achieved the highest ROC-AUC value of 85.9%, and the refined XGBoost model obtained the highest balanced accuracy and weighted F1 of 81% and 81.1%, respectively.

### SMILES notations-embedded deep learning model

#### Model performances

**Figure 5.**
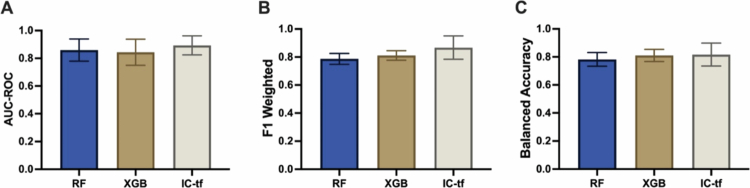
Model performances across three evaluation metrics (A) AUC-ROC, (B) Weighted F1, (C) Balanced Accuracy on the EcGUS inhibitor database with simple supervised RF and XGBoost, and SMILES-embedded IC-tf models. CV = 3. All data were shown as mean ± SD.

All classical supervised ML models rely solely on manually curated features for compound activity prediction, neglecting intrinsic molecular characteristics such as architecture and composition.^21^ ^,^ ^52^ It was argued that accurate and reliable predictions require the incorporation of molecular-level information directly. A recent study also showed that SMILES notations can be applied to deep machine learning.^53^ To address this, a robust classification model that leverages both learned representations from SMILES notations and expert-selected features was developed. Five classification algorithms were used to predict compound activity using the extracted high-dimensional representations. It was shown that the XGBoost modelling with the SMILES notations alone as the input obtained a ROC-AUC value around 70% (Figure S5). This demonstrates that the SMILES notations contribute useful information to enhance model ranking performance. Therefore, this embedded information was further extracted and integrated into a self-attention-based classification model (IC-tf). Improvement in accuracy was observed after integrating embedded information of SMILES notation across three evaluation metrics compared to the classical RF and XGBoost ML models. The ROC-AUC was increased by 3.4% (89.3%, Figure 5A). The balanced accuracy (Figure 5C) and F1-score (Figure 5B) increased to 81.7% and 86.7%, an increase of 0.7 and 5.6 percentage points, respectively, indicating strong discriminative capability and robustness against class imbalance (Table S4). The improvements demonstrated that the SMILES notations encode valuable structural and elemental information that enhances the model’s predictive capabilities.

#### Interpretability of the IC-tf model

**Figure 6.**
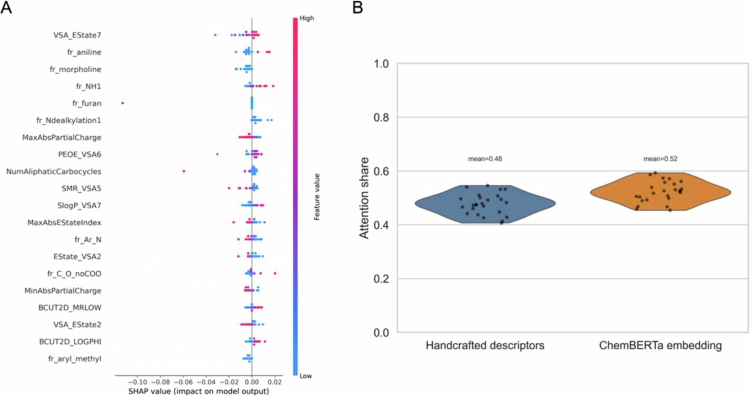
Dual-level interpretability analysis of the IC-tf model. (A) Global SHAP summary plot highlighting the top handcrafted molecular descriptors across the test set. Each point represents a single molecule; the x-axis displays the SHAP value (the feature's directional impact on the prediction), and the color gradient reflects the relative feature value. (B) Global branch-attention distribution. Violin plots illustrate the distribution of transformer attention allocated to the descriptor branch versus the ChemBERTa embedding branch. Overlaid points represent individual samples, with mean attention shares annotated. Because the architecture compresses each modality into a single latent token prior to fusion, attention is strictly interpreted as branch-level reliance rather than feature-level causality.

A dual-level interpretability framework was utilized to elucidate the predictive mechanism of the IC-tf model. First, SHAP was used to identify the handcrafted molecular descriptors with the strongest contributions across the test set (Figure 6A). Many of these features identified by the IC-tf model align consistently with those prioritized by the classical models. In particular, descriptors associated with benzene rings, such as *fr-aniline* and *fr_Ar_N*, were selected, indicating the importance of the cyclic structure. Additionally, van der Waals surface area descriptors (e.g. *VSA_Estate7*) were highlighted, which corroborated the features selected by the Random Forest and the XGBoost models, as well as previous literature.

Second, an analysis of branch-level transformer attention revealed the relative reliance of the model on the handcrafted descriptor branch versus the ChemBERTa embedding branch across test molecules (Figure 6B). Together, these analyses provide complementary interpretations at the descriptor level and the branch level. Importantly, because descriptors and ChemBERTa embeddings are compressed before transformer fusion, attention should be interpreted as branch-level reliance rather than individual feature-level explanation.

### Model evaluation and validation

#### Classification performance analysis

**Figure 7.**
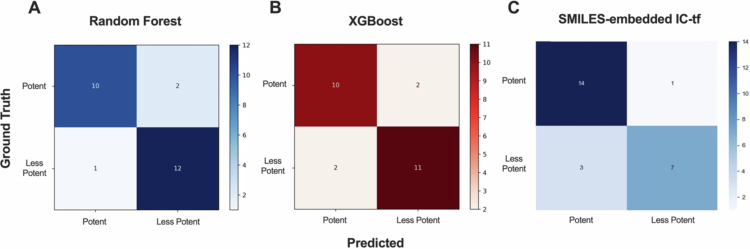
Confusion matrix analysis. (A) Random forest model with feature selection. (B) Classical XGBoost model with feature selection. (C) SMILES-embedded IC-tf model.

The results of the three best-performing models (Classical RF or XGBoost with selective features, and IC-tf) were primarily evaluated using confusion matrices. Across all three models, only three or four inhibitors were misclassified between the potent and less potent categories, while the majority of predictions were accurate (Figure 7). These misclassifications may be attributed to inhibitors with IC_50_ values close to the potency threshold, resulting in prediction confidences of around 50%. This finding underscores the importance of validating model predictions with additional analytical techniques.

#### External model validation

An external database with 20 random compounds, including excipients and flavonoids, was constructed to further validate the accuracy of the basic RF, XGBoost and SMILES-embedded IC-tf models (Table S5). Validation compounds were mainly selected from the Inactive Ingredient for Approved Drug Products database provided by the FDA, based on their functional categories and chemical structures. The inhibitor UNC10201652, which was also present in the original training set, was included as a positive control. The remaining 19 molecules were entirely independent of the training dataset.

The three models categorized the compounds based on whether they would be a potent EcGUS inhibitor with an IC_50_ lower than 1, reporting the probability of being potent. Among the 20 compounds, 18 of them were categorized consistently across the three models, with 2 compounds predicted to be potent and 16 compounds predicted to be less potent. However, the two flavonoids, quercetin and fisetin, were both predicted to be potent by the classical XGB model but less potent by the RF and SMILES-embedded IC-tf models. The probabilities of being potent reported by the basic XGB model were 53.7% for both quercetin and fisetin, while the remaining two models reported the probabilities to be lower than 30% (RF: Quercetin-27.6%, Fisetin-30%; SMILES-embedded IC-tf: Quercetin-11.6%, Fisetin-18.9%).

**Figure 8.**
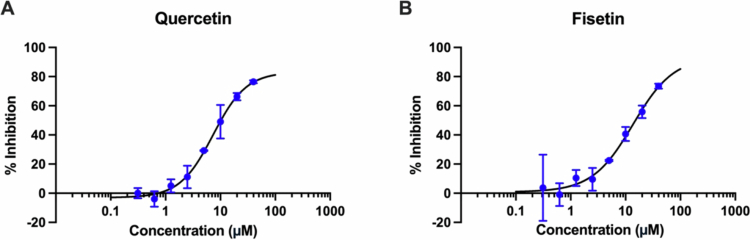
The dose-dependent inhibition curves of quercetin (A) and fisetin (B). All data were shown as mean ± SD.

In order to validate which models had a better performance, the two flavonoids were tested with an *in vitro* EcGUS inhibition assay using PNPG as the probe substrate. To quantitatively characterize the inhibitory effects of quercetin and fisetin against EcGUS, dose-dependent inhibition curves were plotted using different inhibitor concentrations. As shown in Figure 8, quercetin and fisetin inhibit the EcGUS to 80% in a dose-dependent manner. The IC_50_ values of quercetin and fisetin were determined as 7.35 μM and 13.62 μM, respectively, in which they were both less potent inhibitors with IC_50_ values higher than 1 μM. The results are supported by previous research on the inhibitory effect of quercetin on EcGUS, which reported a less potent IC_50_ value.^26^ ^,^ ^54^ The higher IC_50_ value of fisetin is caused by the lack of one hydroxyl group on the pyrogallol group, which is unbeneficial for EcGUS inhibition.^26^ The results of the *in vitro* EcGUS inhibition assay matched the prediction results of the RF and SMILES-embedded IC-tf models, suggesting the better performance these two models can have when predicting inhibitory potency. However, only 20 compounds were included in the external validation set, of which two exhibited inconsistent predictions across the three models. *In vitro* experimental validation is currently limited to these two compounds. Future studies should therefore expand the validation to a larger and more diverse set of candidates to further assess and strengthen the generalizability of the model predictions.

Previous studies have primarily relied on traditional high-throughput screening (HTS) approaches for *in silico* virtual screening of intestinal EcGUS inhibitors.^51^ In comparison, advanced virtual screening techniques, including molecular docking and molecular dynamics (MD) simulations, are computationally demanding when processing large datasets. Machine learning-based HTS is more valuable for reducing the computational burden of molecular docking, particularly in large real-world chemical libraries where low-score compounds constitute the majority and consume substantial computational resources during docking-based virtual screening.^55^ However, *in silico* virtual HTS provides valuable insights into ligand-protein interactions, binding affinities, and conformational stability, which cannot be fully captured by standalone ML or DL models.^56^ Therefore, future approaches should focus on integrating ML predictions with structure-based virtual screening workflows, such as incorporating docking or MD simulations downstream of ML filtering, to further enhance the accuracy and reliability of inhibitor identification.

## Conclusion

In this study, ML and DL were applied to predict the potency of EcGUS inhibitors based on a training database, which included IC_50_ values of 121 EcGUS inhibitors from the literature. Initial exploration demonstrated that potent inhibitors (IC_50_ lower than 1 μM) are more likely to have a molar weight of 200–400 g/mol with moderately branched 2D topology and a TPSA value in the range of 60–80 Å^2^. The best-performing classical ML models consisted of the RF and XGBoost models trained on automatically selected features. The AUC-ROC of the two models in a 3-fold cross-validation were 85.9% (RF) and 84.3% (XGBoost); the f1 scores were 78.7% (RF) and 81.1% (XGBoost); the balanced accuracies were 78.2% (RF) and 81.0% (XGBoost). Further integration of the embedding information of SMILEs notations into a self-attention-based classification model (IC-tf) revealed an improvement in model performance, with an ROC-AUC of 89.3%, a f1 score of 86.7% and a balanced accuracy of 81.7%. The improvement in model performance demonstrated that the 1D features extracted from the SMILES notations can contribute to the prediction. The in-vitro EcGUS inhibition assay, after testing the three models (RF, XGBoost and IC-tf) against a 20-molecule external database, validate the higher accuracy of the RF and IC-tf models in predicting the inhibition potency of quercetin and fisetin. The models developed in this study have been made available for predicting the potency of EcGUS inhibitors, and may be used to digitally screen the suitability of excipients or other drugs for co-administration.

This study highlights the potential of harnessing the power of machine learning to aid the drug discovery pipeline, demonstrating its utility as a proof of concept for digitally screening the suitability of excipients or other drugs for co-administration. However, the models developed in this study have been designed solely for predicting the potency of EcGUS inhibitors. While EcGUS may be the most common and well-established model for studying enzyme-inhibitor interactions, the gut microbiome is an intricate community of trillions of microorganisms, representing a vast array of microbial enzymes. Therefore, further studies incorporating a broader diversity of GUS enzymes and larger sample sizes are required not only to assess and improve the cross-species applicability of the models but also to enhance their stability and predictive performance.

## Supplementary Material

Supplementary Material Supplementary Material

## Data Availability

The optimized predictive models developed in this study are publicly available in the GitHub repository at: https://github.com/Hans0919/ML_EcGUS_Inh.

## References

[1] Weersma RK , Zhernakova A , Fu J . Interaction between drugs and the gut microbiome. Gut. 2020;69:1510–1519. doi: 10.1136/gutjnl-2019-320204. 32409589 PMC7398478

[2] Zimmermann M , Zimmermann-Kogadeeva M , Wegmann R , Goodman AL . Mapping human microbiome drug metabolism by gut bacteria and their genes. Nature. 2019;570:462–467. doi: 10.1038/s41586-019-1291-3. 31158845 PMC6597290

[3] Javdan B , Lopez JG , Chankhamjon P , Lee YJ , Hull R , Wu Q , Wang X , Chatterjee S , Donia MS . Personalized mapping of drug metabolism by the human gut microbiome. Cell. 2020;181:1661–1679 e1622. doi: 10.1016/j.cell.2020.05.001. 32526207 PMC8591631

[4] Pollet RM , D'Agostino EH , Walton WG , Xu Y , Little MS , Biernat KA , Pellock SJ , Patterson LM , Creekmore BC , Isenberg HN , et al. An Atlas of beta-glucuronidases in the human intestinal microbiome. Structure. 2017;25:967–977 e965. doi: 10.1016/j.str.2017.05.003. 28578872 PMC5533298

[5] Gao S , Sun R , Singh R , Yu So S , Chan CT , Savidge T , Hu M . The role of gut microbial beta-glucuronidase in drug disposition and development. Drug Discov Today. 2022;27:103316. doi: 10.1016/j.drudis.2022.07.001. 35820618 PMC9717552

[6] Pellock SJ , Redinbo MR . Glucuronides in the gut: sugar-driven symbioses between microbe and host. J Biol Chem. 2017;292:8569–8576. doi: 10.1074/jbc.R116.767434. 28389557 PMC5448086

[7] Wallace BD , Wang H , Lane KT , Scott JE , Orans J , Koo JS , Venkatesh M , Jobin C , Yeh L , Mani S , et al. Alleviating cancer drug toxicity by inhibiting a bacterial enzyme. Sci. 2010;330:831–835. doi: 10.1126/science.1191175. PMC3110694 21051639

[8] Ervin SM , Hanley RP , Lim L , Walton WG , Pearce KH , Bhatt AP , James LI , Redinbo MR . Targeting regorafenib-induced toxicity through inhibition of gut microbial beta-glucuronidases. ACS Chem Biol. 2019;14:2737–2744. doi: 10.1021/acschembio.9b00663. 31663730 PMC7254866

[9] Cheng KW , Tseng C , Yang C , Tzeng C , Leu Y , Chuang Y , Wang J , Lu Y , Chen Y . Specific inhibition of bacterial beta-glucuronidase by Pyrazolo[4,3-c]quinoline derivatives via a pH-dependent manner to suppress chemotherapy-induced intestinal toxicity. J Med Chem. 2017;60:9222–9238. doi: 10.1021/acs.jmedchem.7b00963. 29120626

[10] Biernat KA , Pellock SJ , Bhatt AP , Bivins MM , Walton WG , Tran BNT , Wei L , Snider MC , Cesmat AP , Tripathy A , et al. Structure, function, and inhibition of drug reactivating human gut microbial beta-glucuronidases. Sci Rep. 2019;9:825. doi: 10.1038/s41598-018-36069-w. 30696850 PMC6351562

[11] Zahid H , Tayara H , Chong KT . Harnessing machine learning to predict cytochrome P450 inhibition through molecular properties. Arch Toxicol. 2024;98:2647–2658. doi: 10.1007/s00204-024-03756-9. 38619593

[12] Grimberg H , Tiwari VS , Tam B , Gur-Arie L , Gingold D , Polachek L , Akabayov B . Machine learning approaches to optimize small-molecule inhibitors for RNA targeting. J Cheminform. 2022;14:4. doi: 10.1186/s13321-022-00583-x. 35109921 PMC8811966

[13] Aykul S , Martinez-Hackert E . Determination of half-maximal inhibitory concentration using biosensor-based protein interaction analysis. Anal Biochem. 2016;508:97–103. doi: 10.1016/j.ab.2016.06.025. 27365221 PMC4955526

[14] Kim S , Chen J , Cheng T , Gindulyte A , He J , Li Q , Shoemaker BA , Thiessen PA , Yu B , Zaslavsky L , et al. PubChem 2025 update. Nucleic Acids Res. 2025;53:D1516–D1525. doi: 10.1093/nar/gkae1059. 39558165 PMC11701573

[15] Veselinovic AM , Veselinovic JB , Zivkovic JV , Nikolic GM . Application of SMILES notation based optimal descriptors in drug discovery and design. Curr Top Med Chem. 2015;15:1768–1779. doi: 10.2174/1568026615666150506151533. 25961525

[16] Lai CH , Kwok APK , Wong KC . Cheminformatic identification of Tyrosyl-DNA phosphodiesterase 1 (Tdp1) inhibitors: a comparative study of SMILES-Based supervised machine learning models. J Pers Med. 2024;14:981. doi: 10.3390/jpm14090981. 39338235 PMC11433629

[17] Patiny L , Zasso M , Kostro D , Bernal A , Castillo AM , Bolaños A , Asencio MA , Pellet N , Todd M , Schloerer N , et al. The C6H6 NMR repository: an integral solution to control the flow of your data from the magnet to the public. Magn Reson Chem. 2018;56:520–528. doi: 10.1002/mrc.4669. 28981966

[18] Eaton EA , Walle UK , Lewis AJ , Hudson T , Wilson AA . Flavonoids, potent inhibitors of the human P-form phenolsulfotransferase. Potential role in drug metabolism and chemoprevention. Drug Metab Dispos. 1996;24:232–237. doi: 10.1016/S0090-9556(25)07302-7. 8742236

[19] Lagunin AA , Romanova MA , Zadorozhny AD , Kurilenko NS , Shilov BV , Pogodin PV , Ivanov SM , Filimonov DA , Poroikov VV . Comparison of quantitative and qualitative (Q)Sar models created for the prediction of K(I) and IC(50) values of antitarget inhibitors. Front Pharmacol. 2018;9:1136. doi: 10.3389/fphar.2018.01136. 30364128 PMC6192375

[20] Alves VM , Borba JVB , Braga RC , Korn DR , Kleinstreuer N , Causey K , Tropsha A , Rua D , Muratov EN . PreS/MD: predictor of sensitization hazard for chemical substances released from medical devices. Toxicol Sci. 2022;189:250–259. doi: 10.1093/toxsci/kfac078. 35916740 PMC9516038

[21] Wang F , Sangfuang N , McCoubrey LE , Yadav V , Elbadawi M , Orlu M , Gaisford S , Basit AW . Advancing oral delivery of biologics: machine learning predicts peptide stability in the gastrointestinal tract. Int J Pharm. 2023;634:122643. doi: 10.1016/j.ijpharm.2023.122643. 36709014

[22] Vamathevan J , Clark D , Czodrowski P , Dunham I , Ferran E , Lee G , Li B , Madabhushi A , Shah P , Spitzer M , et al. Applications of machine learning in drug discovery and development. Nat Rev Drug Discov. 2019;18:463–477. doi: 10.1038/s41573-019-0024-5. 30976107 PMC6552674

[23] de Hond AAH , Steyerberg EW , van Calster B . Interpreting area under the receiver operating characteristic curve. Lancet Digit Health. 2022;4:e853–e855. doi: 10.1016/S2589-7500(22)00188-1. 36270955

[24] McCoubrey LE , Thomaidou S , Elbadawi M , Gaisford S , Orlu M , Basit AW . Machine learning predicts drug metabolism and bioaccumulation by intestinal microbiota. Pharmaceutics. 2021;13:2001. doi: 10.3390/pharmaceutics13122001. 34959282 PMC8707855

[25] Ahmad W , Seyone Chithrananda ES , Grand G , Ramsundar B . ChemBERTa-2: towards chemical foundation models. arXiv. 2022;arXiv:2209.01712 10.48550/arXiv.2209.01712.

[26] Weng ZM , Wang P , Ge G , Dai Z , Wu D , Zou L , Dou T , Zhang T , Yang L , Hou J . Structure-activity relationships of flavonoids as natural inhibitors against *E. coli* beta-glucuronidase. Food Chem Toxicol. 2017;109:975–983. doi: 10.1016/j.fct.2017.03.042. 28347758

[27] Ahlborg UG , Manzoor E , Thunberg T . Inhibition of beta-glucuronidase by chlorinated hydroquinones and benzoquinones. Arch Toxicol. 1977;37:81–87. doi: 10.1007/BF00293856. 327981

[28] Ahmad S , Hughes MA , Lane KT , Redinbo MR , Yeh L , Scott JE . A high throughput assay for discovery of bacterial beta-glucuronidase inhibitors. Curr Chem Genomics. 2011;5:13–20. doi: 10.2174/1875397301105010013. 21643506 PMC3106358

[29] Ahmad S , Hughes MA , Yeh LA , Scott JE . Potential repurposing of known drugs as potent bacterial beta-glucuronidase inhibitors. J Biomol Screen. 2012;17:957–965. doi: 10.1177/1087057112444927. 22535688 PMC8284931

[30] Awolade P , Cele N , Kerru N , Gummidi L , Oluwakemi E , Singh P . Therapeutic significance of beta-glucuronidase activity and its inhibitors: a review. Eur J Med Chem. 2020;187:111921. doi: 10.1016/j.ejmech.2019.111921. 31835168 PMC7111419

[31] Bai Y , Chen L , Cao Y , Hou X , Jia S , Zhou Q , He Y . Beta-glucuronidase inhibition by constituents of mulberry bark. Planta Med. 2021;87:631–641. doi: 10.1055/a-1402-6431. 33733438

[32] Ge Y , Ma Y , Zhao M , Wei J , Wu X , Zhang Z , Yang H , Lei H . Exploring gabosine and chlorogentisyl alcohol derivatives from a marine-derived fungus as EcGUS inhibitors with informatic assisted approaches. Eur J Med Chem. 2022;242:114699. doi: 10.1016/j.ejmech.2022.114699. 36001934

[33] Kawee-ai A , Kim SM . Application of microalgal fucoxanthin for the reduction of colon cancer risk: inhibitory activity of fucoxanthin against β-Glucuronidase and DLD-1 cancer cells. Nat Prod Commun. 2014;9:921–924. 25230493

[34] Pellock SJ , Creekmore BC , Walton WG , Mehta N , Biernat KA , Cesmat AP , Ariyarathna Y , Dunn ZD , Li B , Jin J , et al. Gut microbial beta-glucuronidase inhibition via catalytic cycle interception. ACS Cent Sci. 2018;4:868–879. doi: 10.1021/acscentsci.8b00239. 30062115 PMC6062831

[35] Rao G , Yu H , Zhang M , Cheng Y , Ran K , Wang J , Wei B , Li M , Shan W , Zhan Z , et al. Alpha-glucosidase and bacterial beta-glucuronidase inhibitors from the stems of schisandra sphaerandra staph. Pharmaceuticals (Basel). 2022;15:329. doi: 10.3390/ph15030329. 35337127 PMC8954508

[36] Sun CP , Tian X , Feng L , Wang C , Li J , Huo X , Zhao W , Ning J , Yu Z , Deng S , et al. Inhibition of gut bacterial β-glucuronidase by chemical components from black tea: inhibition interactions and molecular mechanism. Arab J Chem. 2021;14:103457. doi: 10.1016/j.arabjc.2021 ARTN.

[37] Tian XG , Yan J , Sun C , Li J , Ning J , Wang C , Huo X , Zhao W , Yu Z , Feng L , et al. Amentoflavone from *Selaginella tamariscina* as a potent inhibitor of gut bacterial beta-glucuronidase: inhibition kinetics and molecular dynamics stimulation. Chem Biol Interact. 2021;340:109453. doi: 10.1016/j.cbi.2021.109453. 33785314

[38] Wallace BD , Roberts AB , Pollet RM , Ingle JD , Biernat KA , Pellock SJ , Venkatesh MK , Guthrie L , O’Neal SK , Robinson SJ , et al. Structure and inhibition of microbiome beta-glucuronidases essential to the alleviation of cancer drug toxicity. Chem Biol. 2015;22:1238–1249. doi: 10.1016/j.chembiol.2015.08.005. 26364932 PMC4575908

[39] Wang P , Jia Y , Wu R , Chen Z , Yan R . Human gut bacterial beta-glucuronidase inhibition: an emerging approach to manage medication therapy. Biochem Pharmacol. 2021;190:114566. doi: 10.1016/j.bcp.2021.114566. 33865833

[40] Yang F , Zhu W , Sun S , Ai Q , Edirisuriya P , Zhou K . Isolation and structural characterization of specific bacterial beta-glucuronidase inhibitors from noni (*Morinda citrifolia*) fruits. J Nat Prod. 2020;83:825–833. doi: 10.1021/acs.jnatprod.9b00279. 32083868

[41] Yue B , Gao R , Lv C , Yu Z , Wang H , Geng X , Dou W . Berberine improves irinotecan-induced intestinal mucositis without impairing the anti-colorectal cancer efficacy of irinotecan by inhibiting bacterial beta-glucuronidase. Front Pharmacol. 2021;12:774560. doi: 10.3389/fphar.2021.774560. 34795594 PMC8593678

[42] Zi D , Song Y , Lu T , Kise M , Kato A , Wang J , Jia Y , Li Y , Fleet GW , Yu C . Nanomolar beta-glucosidase and beta-galactosidase inhibition by enantiomeric alpha-1-C-alkyl-1,4-dideoxy-1,4-imino-arabinitol derivatives. Eur J Med Chem. 2023;247:115056. doi: 10.1016/j.ejmech.2022.115056. 36603505

[43] Korn EL , Moscow JA , Freidlin B . Dose optimization during drug development: whether and when to optimize. J Natl Cancer Inst. 2023;115:492–497. doi: 10.1093/jnci/djac232. 36534891 PMC10165487

[44] Dashnyam P , Mudududdla R , Hsieh T , Lin T , Chen P , Hsu C . Beta-glucuronidases of opportunistic bacteria are the major contributors to xenobiotic-induced toxicity in the gut. Sci Rep. 2018;8:16372. doi: 10.1038/s41598-018-34678-z. 30401818 PMC6219552

[45] Ghose AK , Viswanadhan VN , Wendoloski JJ . A knowledge-based approach in designing combinatorial or medicinal chemistry libraries for drug discovery. 1. A qualitative and quantitative characterization of known drug databases. J Comb Chem. 1999;1:55–68. doi: 10.1021/cc9800071. 10746014

[46] rdkit/rdkit: 2025_03_3 (Q1 2025) Release v. Release_2025.03.3 (Zenodo, 2025).

[47] McCoubrey LE , Seegobin N , Elbadawi M , Hu Y , Orlu M , Gaisford S , Basit AW . Active machine learning for formulation of precision probiotics. Int J Pharm. 2022;616:121568. doi: 10.1016/j.ijpharm.2022.121568. 35150845

[48] Muniz Castro B , Muñiz Castro B , Elbadawi M , Ong JJ , Pollard T , Song Z , Gaisford S , Pérez G , Basit AW , Cabalar P , et al. Machine learning predicts 3D printing performance of over 900 drug delivery systems. J Control Release. 2021;337:530–545. doi: 10.1016/j.jconrel.2021.07.046. 34339755

[49] Hou T , Zhang W , Xu X . Molecular docking studies of a group of hydroxamate inhibitors with gelatinase-A by molecular dynamics. J Comput Aided Mol Des. 2002;16:27–41. doi: 10.1023/a:1016345810973. 12197664

[50] Buolamwini JK , Raghavan K , Fesen MR , Pommier Y , Kohn KW , Weinstein JN . Application of the electrotopological state index to QSAR analysis of flavone derivatives as HIV-1 integrase inhibitors. Pharm Res. 1996;13:1892–1895. doi: 10.1023/a:1016005813432. 8987091

[51] Cheng TC , Chuang K , Roffler SR , Leu Y , Huang C , Kao C , Hsieh Y , Chang L , Chen C . Discovery of specific inhibitors for intestinal *E. col*i beta-glucuronidase through in silico virtual screening. ScientificWorldJournal. 2015;2015:740815. doi: 10.1155/2015/740815. 25839056 PMC4370192

[52] Barigye SJ , Gomez-Ganau S , Serrano-Candelas E , Gozalbes R . PeptiDesCalculator: software for computation of peptide descriptors. Definition, implementation and case studies for 9 bioactivity endpoints. Proteins. 2021;89:174–184. doi: 10.1002/prot.26003. 32881068

[53] Nuñez-Andrade E , Vidal-Daza I , Ryan JW , Gómez-Bombarelli R , Martin-Martinez FJ . Embedded machine-readable molecular representation for resource-efficient deep learning applications. Digit Discov. 2025;4:776–789. doi: 10.1039/d4dd00230j.

[54] Lin CH , Chou H , Chang C , Chen I , Cheng T , Kuo Y , Ko H . Chemical constituent of beta-glucuronidase inhibitors from the root of *Neolitsea acuminatissima* . Molecules. 2020;25:5170. doi: 10.3390/molecules25215170. 33172041 PMC7664238

[55] Xie Q , Ma W , Zhang J , Li S , Deng X , Xu Y . Exploration on learning molecular docking with deep learning models. Quant Biol. 2023;11:320–331. doi: 10.15302/J-QB-022-0321. 41675244 PMC12807227

[56] Ouassaf M , Mazri R , Khan SU , Rengasamy KRR , Alhatlani BY . Machine learning-guided screening and molecular docking for proposing naturally derived drug candidates against MERS-CoV 3CL protease. Int J Mol Sci. 2025;26:3047. doi: 10.3390/ijms26073047. 40243651 PMC11988297

