# Supplementary material for: High-throughput identification of bacterial β-glucuronidase inhibitors using machine learning
Source: Gut Microbes. 2026 Jun 5;18(1):2681789. doi: 10.1080/19490976.2026.2681789 (PMC13245089; doi:10.1080/19490976.2026.2681789)
Supplement: Supplementary Material [file KGMI_A_2681789_SM0782.docx]

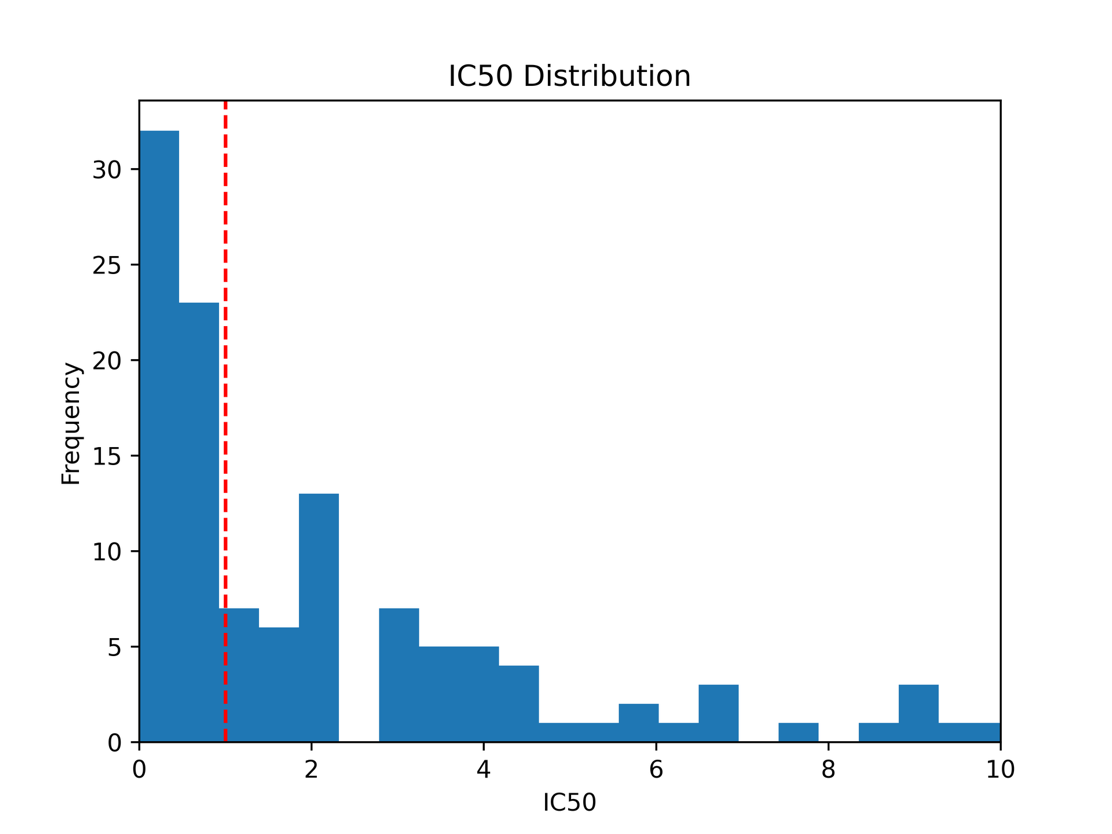
**Supplementary**

**Figure S1**. Distribution of the IC_50_ values across the 122 compounds in the bGUS inhibitor database. IC_50_ = 1 μM is indicated by the red vertical line.


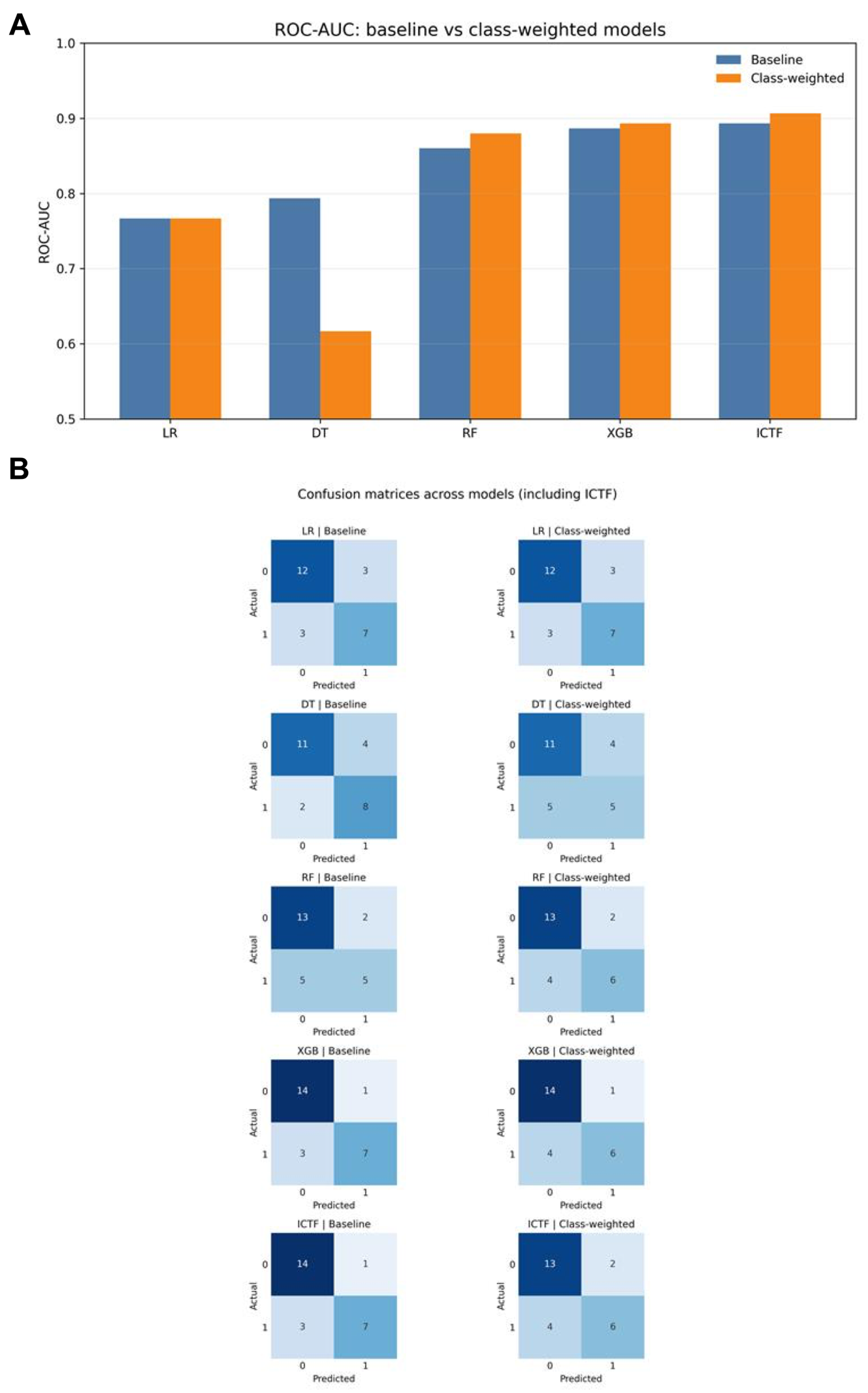


**Figure S2**. Cost-sensitive learning analysis using class-weighted training. (A) ROC-AUC of the baseline and the class-weighted models across 5 different ML models. (B) Confusion matrices across 5 different models.


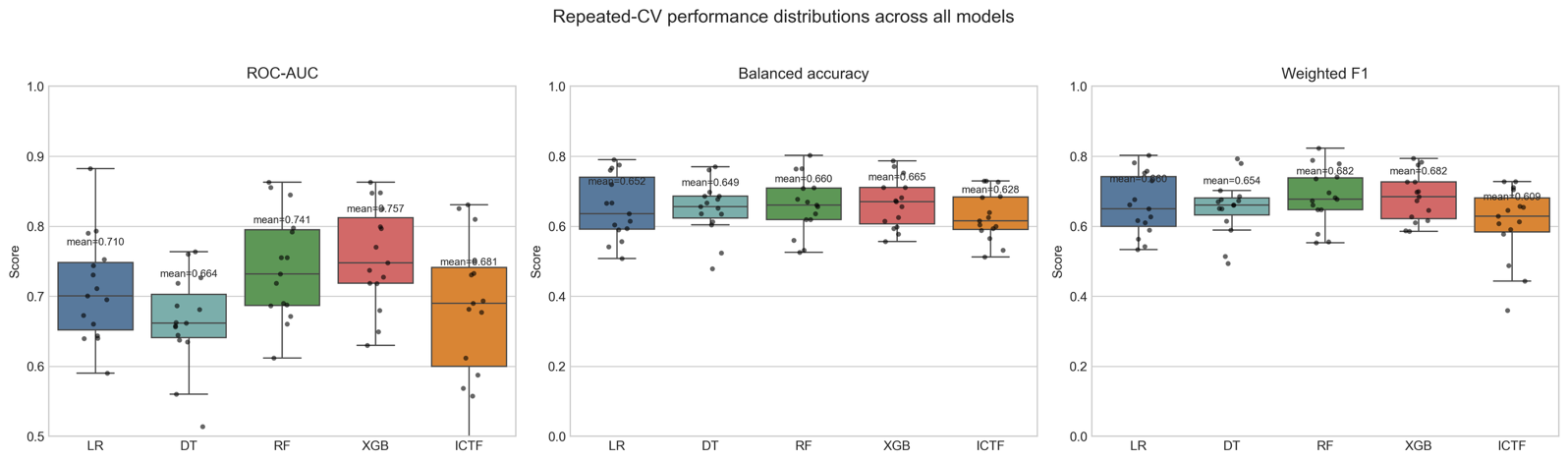


**Figure S3.** Three evaluation metrics for 5× repeated stratified 3-fold cross-validation across five different models with a 60:40 train-test split.


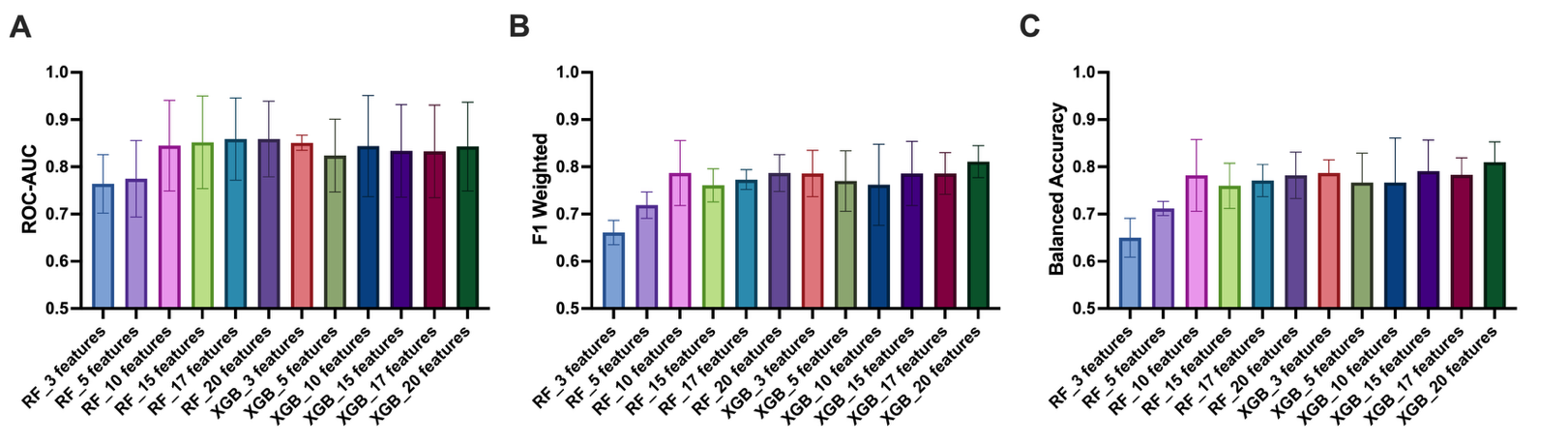


**Figure S4**. Model performances across three evaluation metrics on the simple supervised RF and XGBoost with different numbers of selective features. CV=3. All data were shown as mean ± SD.


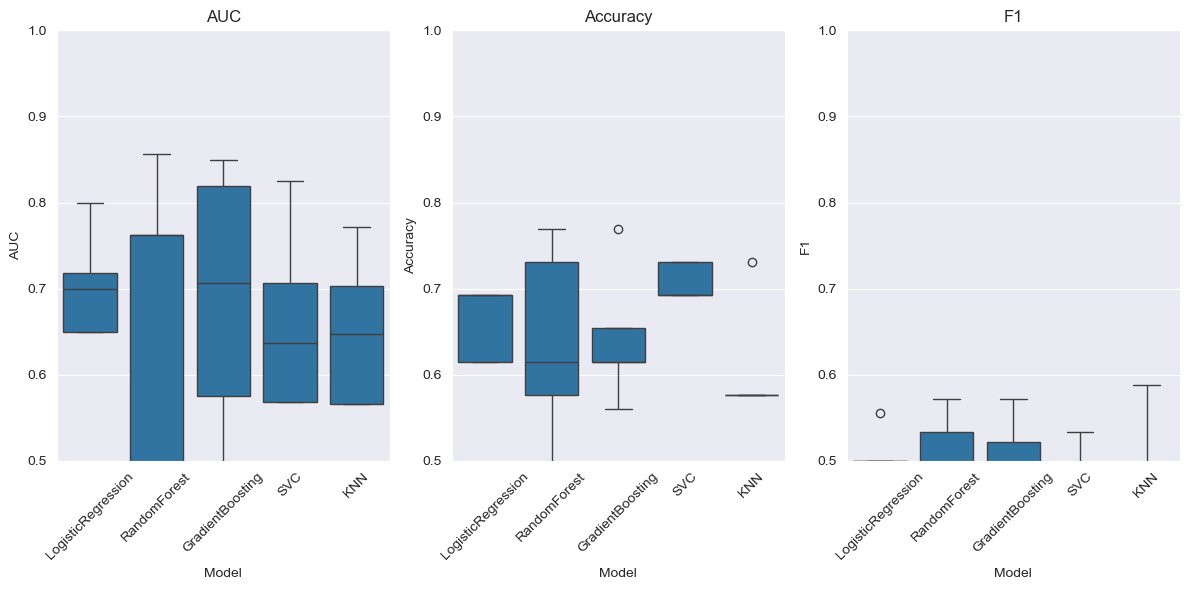


**Figure S5**. Comparative analysis of different embedding strategies. All data were shown as mean ± SD.

**Table S1**. Summary of the 18 publications for the inhibitor database construction

| Index | Author | Year | | Title | Number of Inhibitors | |
| --- | --- | --- | --- | --- | --- | --- |
| 1 | Rao et al. | 2022 | α-Glucosidase and Bacterial β-Glucuronidase Inhibitors from the Stems of Schisandra sphaerandra Staph | | | 1 |
| 2 | Wang et al. | 2021 | Human gut bacterial β-glucuronidase inhibition: An emerging approach to manage medication therapy | | | 43 |
| 3 | Tian et al. | 2021 | Amentoflavone from Selaginella tamariscina as a potent inhibitor of gut bacterial β-glucuronidase: Inhibition kinetics and molecular dynamics stimulation | | | 1 |
| 4 | Yang et al. | 2020 | Isolation and Structural Characterization of Specific Bacterial β-Glucuronidase Inhibitors from Noni (*Morinda citrifolia*) Fruits | | | 4 |
| 5 | Ahlborg et al. | 1977 | Inhibition of β-glucuronidase by chlorinated hydroquinones and benzoquinones | | | 13 |
| 6 | Bai et al. | 2021 | Beta-Glucuronidase Inhibition by Constituents of Mulberry Bark | | | 5 |
| 7 | Ahmad et al. | 2012 | Potential repurposing of known drugs as potent bacterial β-glucuronidase inhibitors | | | 6 |
| 8 | Kawee-Ai, A., & Kim, S. M. | 2014 | Application of microalgal fucoxanthin for the reduction of colon cancer risk: inhibitory activity of fucoxanthin against beta-glucuronidase and DLD-1 cancer cells | | | 1 |
| 9 | Pellock et al. | 2018 | Gut Microbial β-Glucuronidase Inhibition via Catalytic Cycle Interception | | | 3 |
| 10 | Cheng et al. | 2017 | Specific Inhibition of Bacterial β-Glucuronidase by Pyrazolo[4,3-c]quinoline Derivatives via a pH-Dependent Manner To Suppress Chemotherapy-Induced Intestinal Toxicity | | | 11 |
| 11 | Sun et al. | 2021 | Inhibition of gut bacterial β-glucuronidase by chemical components from black tea: Inhibition interactions and molecular mechanism | | | 4 |
| 12 | Yue et al. | 2021 | Berberine Improves Irinotecan-Induced Intestinal Mucositis Without Impairing the Anti-colorectal Cancer Efficacy of Irinotecan by Inhibiting Bacterial β-glucuronidase | | | 1 |
| 13 | Ahmad et al. | 2012 | Potential repurposing of known drugs as potent bacterial β-glucuronidase inhibitors | | | 6 |
| 14 | Awolade, P. et al. | 2020 | Therapeutic significance of β-glucuronidase activity and its inhibitors: A review | | | 9 |
| 15 | Wallace, B. D. et al. | 2015 | Structure and Inhibition of Microbiome β-Glucuronidases Essential to the Alleviation of Cancer Drug Toxicity | | | 1 |
| 16 | Ge, Y. et al. | 2022 | Exploring gabosine and chlorogentisyl alcohol derivatives from a marine-derived fungus as EcGUS inhibitors with informatic assisted approaches | | | 14 |
| 17 | Zi, D. et al. | 2022 | Nanomolar β-glucosidase and β-galactosidase inhibition by enantiomeric α-1-C-alkyl-1,4-dideoxy-1,4-imino-arabinitol derivatives | | | 3 |
| 18 | Weng, Z. M. et al. | 2017 | Structure-activity relationships of flavonoids as natural inhibitors against E. coli β-glucuronidase | | | 4 |

**Table S2**. Sensitivity analysis of model performance under alternative IC_50_ thresholds

| **IC50 Threshold** | **% Potent** | **Model** | **ROC-AUC** | **Weighted F1** | **Balanced Accuracy** |
| --- | --- | --- | --- | --- | --- |
| 0.5 μM | 22% | RF | 0.800 | 0.807 | 0.707 |
| 1 μM | 40% | RF | 0.829 | 0.760 | 0.760 |
| 5 μM | 78.70% | RF | 0.764 | 0.74 | 0.607 |
| 0.5 μM | 22% | XGBoost | 0.787 | 0.816 | 0.715 |
| 1 μM | 40% | XGBoost | 0.828 | 0.812 | 0.804 |
| 5 μM | 78.70% | XGBoost | 0.813 | 0.783 | 0.678 |

**Table S3**. Index number (#) and feature names used in the full feature set.

| # | Feature name | # | Feature name | # | Feature name | # | Feature name | # | Feature name |
| --- | --- | --- | --- | --- | --- | --- | --- | --- | --- |
| 0 | MaxEStateIndex | 42 | Kappa2 | 84 | EState_VSA11 | 126 | fr_ArN | 168 | fr_hdrzine |
| 1 | MinEStateIndex | 43 | Kappa3 | 85 | EState_VSA2 | 127 | fr_Ar_COO | 169 | fr_hdrzone |
| 2 | MaxAbsEStateIndex | 44 | LabuteASA | 86 | EState_VSA3 | 128 | fr_Ar_N | 170 | fr_imidazole |
| 3 | MinAbsEStateIndex | 45 | PEOE_VSA1 | 87 | EState_VSA4 | 129 | fr_Ar_NH | 171 | fr_imide |
| 4 | qed | 46 | PEOE_VSA10 | 88 | EState_VSA5 | 130 | fr_Ar_OH | 172 | fr_isocyan |
| 5 | MolWt | 47 | PEOE_VSA11 | 89 | EState_VSA6 | 131 | fr_COO | 173 | fr_isothiocyan |
| 6 | HeavyAtomMolWt | 48 | PEOE_VSA12 | 90 | EState_VSA7 | 132 | fr_COO2 | 174 | fr_ketone |
| 7 | ExactMolWt | 49 | PEOE_VSA13 | 91 | EState_VSA8 | 133 | fr_C_O | 175 | fr_ketone_Topliss |
| 8 | NumValenceElectrons | 50 | PEOE_VSA14 | 92 | EState_VSA9 | 134 | fr_C_O_noCOO | 176 | fr_lactam |
| 9 | NumRadicalElectrons | 51 | PEOE_VSA2 | 93 | VSA_EState1 | 135 | fr_C_S | 177 | fr_lactone |
| 10 | MaxPartialCharge | 52 | PEOE_VSA3 | 94 | VSA_EState10 | 136 | fr_HOCCN | 178 | fr_methoxy |
| 11 | MinPartialCharge | 53 | PEOE_VSA4 | 95 | VSA_EState2 | 137 | fr_Imine | 179 | fr_morpholine |
| 12 | MaxAbsPartialCharge | 54 | PEOE_VSA5 | 96 | VSA_EState3 | 138 | fr_NH0 | 180 | fr_nitrile |
| 13 | MinAbsPartialCharge | 55 | PEOE_VSA6 | 97 | VSA_EState4 | 139 | fr_NH1 | 181 | fr_nitro |
| 14 | FpDensityMorgan1 | 56 | PEOE_VSA7 | 98 | VSA_EState5 | 140 | fr_NH2 | 182 | fr_nitro_arom |
| 15 | FpDensityMorgan2 | 57 | PEOE_VSA8 | 99 | VSA_EState6 | 141 | fr_N_O | 183 | fr_nitro_arom_nonortho |
| 16 | FpDensityMorgan3 | 58 | PEOE_VSA9 | 100 | VSA_EState7 | 142 | fr_Ndealkylation1 | 184 | fr_nitroso |
| 17 | BCUT2D_MWHI | 59 | SMR_VSA1 | 101 | VSA_EState8 | 143 | fr_Ndealkylation2 | 185 | fr_oxazole |
| 18 | BCUT2D_MWLOW | 60 | SMR_VSA10 | 102 | VSA_EState9 | 144 | fr_Nhpyrrole | 186 | fr_oxime |
| 19 | BCUT2D_CHGHI | 61 | SMR_VSA2 | 103 | FractionCSP3 | 145 | fr_SH | 187 | fr_para_hydroxylation |
| 20 | BCUT2D_CHGLO | 62 | SMR_VSA3 | 104 | HeavyAtomCount | 146 | fr_aldehyde | 188 | fr_phenol |
| 21 | BCUT2D_LOGPHI | 63 | SMR_VSA4 | 105 | NHOHCount | 147 | fr_alkyl_carbamate | 189 | fr_phenol_noOrthoHbond |
| 22 | BCUT2D_LOGPLOW | 64 | SMR_VSA5 | 106 | NOCount | 148 | fr_alkyl_halide | 190 | fr_phos_acid |
| 23 | BCUT2D_MRHI | 65 | SMR_VSA6 | 107 | NumAliphaticCarbocycles | 149 | fr_allylic_oxid | 191 | fr_phos_ester |
| 24 | BCUT2D_MRLOW | 66 | SMR_VSA7 | 108 | NumAliphaticHeterocycles | 150 | fr_amide | 192 | fr_piperdine |
| 25 | BalabanJ | 67 | SMR_VSA8 | 109 | NumAliphaticRings | 151 | fr_amidine | 193 | fr_piperzine |
| 26 | BertzCT | 68 | SMR_VSA9 | 110 | NumAromaticCarbocycles | 152 | fr_aniline | 194 | fr_priamide |
| 27 | Chi0 | 69 | SlogP_VSA1 | 111 | NumAromaticHeterocycles | 153 | fr_aryl_methyl | 195 | fr_prisulfonamd |
| 28 | Chi0n | 70 | SlogP_VSA10 | 112 | NumAromaticRings | 154 | fr_azide | 196 | fr_pyridine |
| 29 | Chi0v | 71 | SlogP_VSA11 | 113 | NumHAcceptors | 155 | fr_azo | 197 | fr_quatN |
| 30 | Chi1 | 72 | SlogP_VSA12 | 114 | NumHDonors | 156 | fr_barbitur | 198 | fr_sulfide |
| 31 | Chi1n | 73 | SlogP_VSA2 | 115 | NumHeteroatoms | 157 | fr_benzene | 199 | fr_sulfonamd |
| 32 | Chi1v | 74 | SlogP_VSA3 | 116 | NumRotatableBonds | 158 | fr_benzodiazepine | 200 | fr_sulfone |
| 33 | Chi2n | 75 | SlogP_VSA4 | 117 | NumSaturatedCarbocycles | 159 | fr_bicyclic | 201 | fr_term_acetylene |
| 34 | Chi2v | 76 | SlogP_VSA5 | 118 | NumSaturatedHeterocycles | 160 | fr_diazo | 202 | fr_tetrazole |
| 35 | Chi3n | 77 | SlogP_VSA6 | 119 | NumSaturatedRings | 161 | fr_dihydropyridine | 203 | fr_thiazole |
| 36 | Chi3v | 78 | SlogP_VSA7 | 120 | RingCount | 162 | fr_epoxide | 204 | fr_thiocyan |
| 37 | Chi4n | 79 | SlogP_VSA8 | 121 | MolLogP | 163 | fr_ester | 205 | fr_thiophene |
| 38 | Chi4v | 80 | SlogP_VSA9 | 122 | MolMR | 164 | fr_ether | 206 | fr_unbrch_alkane |
| 39 | HallKierAlpha | 81 | TPSA | 123 | fr_Al_COO | 165 | fr_furan | 207 | fr_urea |
| 40 | Ipc | 82 | EState_VSA1 | 124 | fr_Al_OH | 166 | fr_guanido |  |  |
| 41 | Kappa1 | 83 | EState_VSA10 | 125 | fr_Al_OH_noTert | 167 | fr_halogen |  |  |

**Table S4**. Different model performances on the EcGUS inhibitor database with full feature set and selected feature set (CV=3). Std: standard deviation.

| # Features | Model | ROC-AUC | ROC-AUC Std | Balanced accuracy | Balanced accuracy Std | Weighted F1 | Weighted F1 std |
| --- | --- | --- | --- | --- | --- | --- | --- |
| 208 | ExtraTree | 0.815 | 0.035 | 0.759 | 0.042 | 0.76 | 0.035 |
| 208 | RF | 0.829 | 0.065 | 0.76 | 0.021 | 0.76 | 0.004 |
| 208 | DT | 0.763 | 0.016 | 0.757 | 0.023 | 0.749 | 0.019 |
| 208 | kNN_1 | 0.707 | 0.033 | 0.707 | 0.033 | 0.683 | 0.033 |
| 208 | kNN_2 | 0.732 | 0.033 | 0.681 | 0.038 | 0.637 | 0.036 |
| 208 | kNN_4 | 0.73 | 0.071 | 0.652 | 0.075 | 0.629 | 0.066 |
| 208 | XGBoost | 0.828 | 0.057 | 0.804 | 0.062 | 0.812 | 0.052 |
| 208 | LR_lasso | 0.601 | 0.074 | 0.689 | 0.075 | 0.682 | 0.087 |
| 208 | LR_ridge | 0.646 | 0.031 | 0.677 | 0.063 | 0.68 | 0.066 |
|  | Baseline | 0.5 | - | 0.613 | 0.019 | 0.467 | 0.023 |
| SELECTED FEATURES | | | | | | | |
| 20 | RF | 0.859 | 0.08 | 0.782 | 0.049 | 0.787 | 0.039 |
| 20 | XGBoost | 0.843 | 0.094 | 0.81 | 0.043 | 0.811 | 0.034 |
| 15 | DT | 0.763 | 0.016 | 0.715 | 0.074 | 0.731 | 0.07 |
| 20 | LR | 0.798 | 0.084 | 0.751 | 0.071 | 0.76 | 0.057 |
| 32* | IC-tf | 0.893 | 0.068 | 0.8167 | 0.0819 | 0.8669 | 0.0832 |

* 32 features were selected from molecular embeddings and physicochemical feature embeddings.

**Table S5.** External validation database with 20 compounds

| **Compound** | **SMILEs** | **Category** |
| --- | --- | --- |
| Alpha-Tocopherol Acetate | CC1=C(C(=C(C2=C1O[C@](CC2)(C)CCC[C@H](C)CCC[C@H](C)CCCC(C)C)C)OC(=O)C)C | Vitamin |
| C.I. Food Yellow 10 | C1=CC=C(C=C1)N=NC2=C(C=CC3=CC=CC=C32)N | Food Colorant |
| Acesulfame | CC1=CC(=O)NS(=O)(=O)O1 | Sweetener |
| Acetyl Tributyl Citrate | CCCCOC(=O)CC(CC(=O)OCCCC)(C(=O)OCCCC)OC(=O)C | Plasticiser |
| [2-(Methacryloyloxy)ethyl]dimethyl-(3-sulfopropyl)ammonium hydroxide | CC(=C)C(=O)OCC[N+](C)(C)CCCS(=O)(=O)[O-] | Drug coatings |
| Ethylene glycol dimethacrylate | CC(=C)C(=O)OCCOC(=O)C(=C)C | Drug coatings |
| Quercetin | C1=CC(=C(C=C1C2=C(C(=O)C3=C(C=C(C=C3O2)O)O)O)O)O | Flavonoids |
| Syringaldehyde | COC1=CC(=CC(=C1O)OC)C=O | Fragrance agent |
| Tween 80 | CCCCCCCCC=CCCCCCCCC(=O)OCCOCC(C1C(CC(O1)OCCO)OCCO)OCCO | Solubilizer |
| Lactose monohydrate | C([C@@H]1[C@@H]([C@@H]([C@H]([C@@H](O1)O[C@@H]2[C@H](O[C@@H]([C@@H]([C@H]2O)O)O)CO)O)O)O)O.O | Sweetener |
| Hydroxyethyl cellulose | CC1C(C(C(C(O1)CO)OC2C(C(C(C(O2)COC3C(C(C(C(O3)CO)OC)O)O)OC4C(C(C(C(O4)CO)OC)O)OCCO)O)O)O)O | Gelling agent |
| Isomalt | C([C@@H]1[C@H]([C@@H]([C@H]([C@H](O1)OC[C@H]([C@H]([C@@H](C(CO)O)O)O)O)O)O)O)O | Sweetener |
| Ponceau4R | C1=CC=C2C(=C1)C(=CC=C2S(=O)(=O)[O-])N=NC3=C(C=CC4=CC(=CC(=C43)S(=O)(=O)[O-])S(=O)(=O)[O-])O | Food Colorant |
| Sunset Yellow | C1=CC(=CC=C1N=NC2=C(C=CC3=C2C=CC(=C3)S(=O)(=O)[O-])O)S(=O)(=O)[O-] | Food Colorant |
| Brilliant Blue R | C1=CC=C2C(=C1)C(=O)C3=C(C2=O)C(=C(C=C3NC4=CC(=CC=C4)S(=O)(=O)CCOS(=O)(=O)[O-])S(=O)(=O)[O-])N | Food Colorant |
| Allure red | COc3cc(c(C)cc3/N=N/c1c2ccc(cc2ccc1O)S([O-])(=O)=O)S([O-])(=O)=O | Food Colorant |
| Brilliant Black | CC(=O)NC1=C2C(=C(C=C1)S(=O)(=O)[O-])C=C(C(=NNC3=C4C=C(C=CC4=C(C=C3)N=NC5=CC=C(C=C5)S(=O)(=O)[O-])S(=O)(=O)[O-])C2=O)S(=O)(=O)[O-] | Food Colorant |
| demethoxydaibucarboline A | OC4CCC(CC2NCCC3C1CC(O)CCC1[NH]C23)CC4 | Inhibitor w/o IC_50_ |
| Fisetin | C1=CC(=C(C=C1C2=C(C(=O)C3=C(O2)C=C(C=C3)O)O)O)O | Flavonoids |
| UNC10201652 | C1CCC2=C(C1)C3=C(N=C2N4CCOCC4)SC5=C3N=NN=C5N6CCNCC6 | Control |
